# Successful endoscopic management of post-coloplasty cervical esophagocutaneous fistula using sequential diabolo and fully covered stents

**DOI:** 10.1055/a-2772-5256

**Published:** 2026-01-22

**Authors:** Abdeldjalil Sais, Jérôme Rivory, Florian Rostain, Alexandru Lupu, Marius Baldir, Jean Grimaldi, Mathieu Pioche

**Affiliations:** 1 639305 Department of Gastroenterology and Endoscopy, Groupement Hospitalier Portes de Provence (GHPP), Montélimar, France; 2 Department of Gastroenterology and Endoscopy, Hôpital Edouard Herriot, Hospices Civils de Lyon, Lyon, France; 3 36609 Department of Emergency and General Surgery, Hôpital Edouard Herriot, Hospices Civils de Lyon, Lyon, France

A 23-year-old woman with a complex psychiatric history—including multiple suicide attempts and ingestion of caustic agents—presented after deliberate ingestion of 200 mL of corrosive liquid. Initial computed tomography and emergent upper endoscopy revealed necrosis of the esophageal mucosa and extensive gastric injury. She underwent total esogastrectomy with cervical esophagostomy and jejunostomy feeding.

Over the following months, she developed a severe, recurrent stenosis of the cervical esophagostomy, causing salivary obstruction, aspiration, and repeated respiratory complications. Several retrograde endoscopic dilatations (4–11 mm) were performed using biliary balloons, allowing only partial functional improvement.

She underwent retrosternal coloplasty with cervical colo-esophageal anastomosis to restore continuity. The postoperative course was complicated by an anastomotic dehiscence, resulting in a large cervical esophagocutaneous fistula and complete stenosis of the colonic conduit. Surgical revision with the placement of a T-tube drain was required ( Fig. 1 ).

**Fig. 1:**
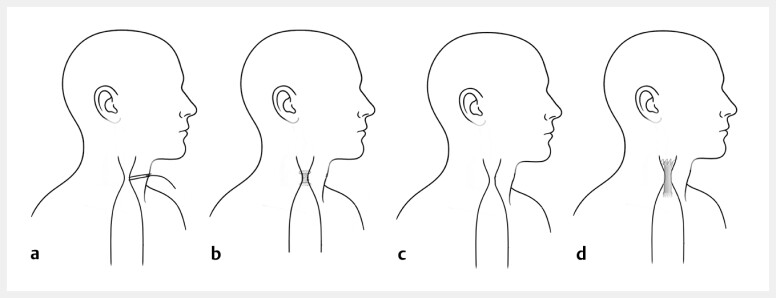
Endoscopic management of a post-coloplasty cervical fistula and stricture. **a** Cervical esophagocutaneous fistula with a T-tube drain. **b** Placement of a short diabolo stent for the fistula sealing and stricture support. **c** Fistula closed with the persistent severe stricture. **d** A fully covered esophageal stent fixed with sutures to prevent migration.

Endoscopic management of a post-coloplasty cervical esophagocutaneous fistula, using a diabolo stent for fistula, followed by a fully covered esophageal stent secured with endoscopic sutures.Video 1

Re-evaluation showed persistent fistula output and worsening dysphagia. Endoscopy demonstrated a tight anastomotic stricture with direct communication to the cervical cutaneous cavity. The T-tube was removed and a 40-mm × 16-mm “diabolo” stent was placed to simultaneously cover the fistula tract and support the anastomosis ( Video 1 ). Four weeks later, follow-up endoscopy confirmed the complete closure of the fistula but persistent anastomotic stenosis. The diabolo stent was removed, and a fully covered 24-mm × 110-mm esophageal stent was deployed and endoscopically sutured with SutuArt (Olympus) to prevent migration.

At a 2-month follow-up and after removing the fully covered stent, imaging and endoscopic assessment confirmed a healed fistula and stable anastomosis.

This case illustrates that the sequential placement of a diabolo stent followed by a sutured fully covered stent can allow the effective management of complex cervical post-coloplasty fistulas and strictures, avoiding further surgical intervention 1 2 3 .

Endoscopy_UCTN_Code_TTT_1AO_2AI

## References

[1] Oprisanescu D Bucur D Sandru V Endoscopic treatment of benign esophageal fistulas using fully-covered metallic esophageal stents Chirurgia (Bucur) 2018 113 108 115 10.21614/chirurgia.113.1.108 29509537

[2] van Boeckel PGA Dua KS Weusten BLAM Fully covered self-expandable metal stents (SEMS), partially covered SEMS and self-expandable plastic stents for the treatment of benign esophageal ruptures and anastomotic leaks BMC Gastroenterol 2012 12 19 22375711 10.1186/1471-230X-12-19 PMC3313862

[3] Wu G Yin M Zhao YS Novel esophageal stent for treatment of cervical anastomotic leakage after esophagectomy Surg Endosc 2017 31 5024 5031 10.1007/s00464-017-5545-6 28432462

